# Variable Behavior of iPSCs Derived from CML Patients for Response to TKI and Hematopoietic Differentiation

**DOI:** 10.1371/journal.pone.0071596

**Published:** 2013-08-23

**Authors:** Aurélie Bedel, Jean- Max Pasquet, Éric Lippert, Miguel Taillepierre, Valérie Lagarde, Sandrine Dabernat, Pierre Dubus, Lucie Charaf, François Beliveau, Hubert de Verneuil, Emmanuel Richard, François-Xavier Mahon, François Moreau-Gaudry

**Affiliations:** 1 Inserm U1035, Biothérapies des maladies génétiques et cancers, Bordeaux, France; 2 Université Bordeaux, EA 2406, Bordeaux, France; 3 Université Bordeaux Segalen, Bordeaux, France; B.C. Cancer Agency, Canada

## Abstract

Chronic myeloid leukemia disease (CML) found effective therapy by treating patients with tyrosine kinase inhibitors (TKI), which suppress the BCR-ABL1 oncogene activity. However, the majority of patients achieving remission with TKI still have molecular evidences of disease persistence. Various mechanisms have been proposed to explain the disease persistence and recurrence. One of the hypotheses is that the primitive leukemic stem cells (LSCs) can survive in the presence of TKI. Understanding the mechanisms leading to TKI resistance of the LSCs in CML is a critical issue but is limited by availability of cells from patients. We generated induced pluripotent stem cells (iPSCs) derived from CD34^+^ blood cells isolated from CML patients (CML-iPSCs) as a model for studying LSCs survival in the presence of TKI and the mechanisms supporting TKI resistance. Interestingly, CML-iPSCs resisted to TKI treatment and their survival did not depend on BCR-ABL1, as for primitive LSCs. Induction of hematopoietic differentiation of CML-iPSC clones was reduced compared to normal clones. Hematopoietic progenitors obtained from iPSCs partially recovered TKI sensitivity. Notably, different CML-iPSCs obtained from the same CML patients were heterogeneous, in terms of BCR-ABL1 level and proliferation. Thus, several clones of CML-iPSCs are a powerful model to decipher all the mechanisms leading to LSC survival following TKI therapy and are a promising tool for testing new therapeutic agents.

## Introduction

Chronic myeloid leukemia (CML) is a model of hematopoietic stem cell (HSC) disorder driven by the Philadelphia chromosome (Ph) and the *BCR-ABL1* gene. The tyrosine kinase inhibitors (TKI), by suppressing the BCR-ABL1 oncogene activity, are efficient in treating CML. However, the majority of patients reaching remission with TKI still have the molecular evidence of disease persistence, and treatment cessation often leads to molecular relapses [1]–[4]. One of the hypotheses is that the primitive leukemic stem cells (LSCs) can survive in the presence of TKI [5]–[7]. Little is known about the resistance of CML-LSCs. Recent studies indicate that their survival could be BCR-ABL1-independent [1], [6]. To eliminate them permanently, it is crucial to better understand the mechanisms leading to their persistence. However, these cells are by nature very rare and poorly characterized at the molecular level, rendering the exploration of signaling pathways and the identification of new therapeutic agents very challenging.

The discovery of key transcription factors enabling reprogramming a somatic cell into a pluripotent stem cell, called induced pluripotent stem cell (iPSC) open new avenues in medicine [8]–[11]. Since iPSCs can be maintained indefinitely *in vitro*, they represent an unlimited source of cells, which could overcome the difficulty of obtaining sufficient amounts of LSCs in the chronic phase of CML. Thus, iPSCs become an attractive model for cancer stem cell studying, especially the LSC behavior and the screening of new therapeutic targets reducing LSC survival.

We generated iPSCs derived from CD34^+^ blood cells isolated from two CML patients (CML-iPSCs) to produce high numbers of CML-LSCs. We observed heterogeneity between the CML-iPSC clones in terms of BCR-ABL1 level and proliferation in presence of TKI.

## Materials and Methods

### Ethics Statement

Written informed consents were obtained in accordance with the Declaration of Helsinki from all participants and data were analyzed anonymously. The use of CB sample was approved by the local Institutional Review Board of “*Maison de Santé de Bagatelle”* (Talence, France). The study was approved by the local Ethics Committee *“Comité Consultatif de Protection des Personnes dans la Recherche Biomédicale*” (CCPRB) de Bordeaux at the University of Bordeaux.

### Human iPSC generation from CD34^+^ cells (cord blood and CML patients)

Primary CD34^+^ cells were isolated from a cord blood (CB), and from two CML peripheral blood (PB) collected at the diagnosis (2 patients in chronic phase with Major Molecular Response after 6-month-imatinib-treatment). Briefly, mononuclear cells were isolated by Ficoll gradient. CD34^+^ cells were purified according to the manufacturer's instructions (Miltenyi Biotech) and purity was analyzed by flow cytometry using phycoerythrin-conjugated anti-CD34 antibody (Becton Dickinson). Cryopreserved CD34^+^ cells were thawed and cultured 2 days in expansion medium consisting in Stem Span SFEM (Stem cell Technologies, Grenoble, France) supplemented with Flt3-L (50 ng/mL), SCF (50 ng/mL) and human TPO (50 ng/mL) (all from Peprotech, Rocky Hill, NJ, USA). iPSCs generation were obtained by transduction of CD34^+^ cells with the two excisable SIN-lentivectors OSK1 and Mshp53 (flanked by LoxP sites) at a multiplicity of infection (MOI) of 100 [12]. After an additional 2 day-culture in the same expansion medium, cells were transferred onto mitomycined mouse embryonic fibroblasts (MEFs) and cultured in ES medium as described below. Starting from day 14 to 22, the individual iPSC colonies were picked up for expansion.

### Human iPSC culture

Human iPSC clones were maintained as undifferentiated cells in cocultures with mitomycined MEFs (Embryomax©, Primary Mouse Embryonic fibroblasts, strain CF1, Millipore). The ES medium used was: KO-DMEM (Invitrogen, Villebon sur Yvette, France) containing 20% KOSR (Invitrogen) (vol/vol), 15 ng/mL human bFGF (Peprotech), 1 mM GlutaMAX^TM^ (Invitrogen), 100 μM Non-Essential Amino Acids (Invitrogen), 100 μM 2-mercaptoethanol (Sigma-Aldrich, Saint Louis, MO, USA), 50 µg/mL ascorbic acid (Sigma-Aldrich), 0.5 mM butyrate sodium (Sigma-Aldrich), 50 U/mL penicillin and 50 mg/mL streptomycin (Invitrogen). The ES medium was changed every day.

### iPSC characterization

Immunofluorescence staining: to detect pluripotency markers, cells grown in 24-well plates were fixed by 4% paraformaldehyde and permeabilized with ice-cold 0.2% Triton X-100 in PBS. After saturation with PBS-triton 0.2%-HSA 1%, cells were stained with primary antibodies for 1 hour followed by incubation with a second fluorochrome-labeled antibody (Alexa Fluor, Invitrogen). Primary antibodies used were: OCT4 (clone C-10, Santa Cruz,CA, USA), SOX2 (Abcam, Cambridge, UK), KLF4 (Abcam), NANOG (Abcam), SSEA-4 (clone 813–70, Stem Cell technologies), and TRA1-60 (Stem Cell technologies).

For teratoma induction, iPSCs were plated in a 10-cm MEFs feeder dish. At day 6, approximately 2×10^6^ cells were harvested, resuspended in 100 µL of ES medium containing 10 μM of the Rho-associated kinase (Rock) inhibitor Y-27632 (Sigma) and injected into NOD-SCID *IL2Rg*-null (NSG) mice (subcutaneous space). The NSG mice were produced and housed in the Bordeaux University animal facility. This study was carried out in strict accordance with the recommendation of *“le comité d*'*éthique de Bordeaux en experimentation animale”* (Institutional Animal Care and Use Committee) and approved by it (agreement number is A33063916). Animals were included in protocols between the age of 6 and 8 weeks. Teratomas were harvested 8 to 12 weeks after injection. Paraffin-embedded tissue was sliced and stained with alcian blue.

### Karyotyping

After synchronization with FrdU followed by a thymidine chase, standard R-banding analysis was performed on metaphases obtained with all iPSC clones. At least 20 metaphases were fully karyotyped.

### Western-blot and qRT-PCR analysis

Protein lysates were prepared according to Gioia et al. [13] Protein concentration was measured by the BCA^TM^ Protein Assay (Pierce, USA) and lysates were stored at −80°C. Approximately 25 µg of proteins were resolved on 10% SDS-PAGE gels, transferred onto PVDF membranes (BIO-RAD, France) by semi-dry electrophoretic transfer, probed with individual antibodies, and visualized by the ECL system (Perkin Elmer, France). The following antibodies were used: anti-ABL1 (8E9) from Becton-Dickinson (France), anti-pTyr (4G10) from Millipore (France), anti-CRKL (C-20) and anti-HSP60 (K-19) from Santa Cruz (Germany), and anti-pCRKL, anti-STAT3 and anti-pSTAT3 from Cell signaling (France). p210 *BCR-ABL1* expression was down-regulated through the lentiviral expression of shRNA (shBCR-ABL1) as described earlier [14]. The shRNA negative control lentiviral vector (shC) targets the DSRed gene that is absent in our cells.

Determination of *BCR-ABL1/ABL1* ratios by qRT-PCR was performed as previously described by Mahon FX et al. [3].

### TKI test on iPSC survival

IPSCs were dissociated into single cells with accutase (Stem Cell Technologies) and plated at 10,000 cells per well in 12-well MEFs plates with ES medium in presence of ROCK inhibitor. At day 5, iPSC lines were incubated for 6 days in the presence or absence of TKI (imatinib 1 to 20 µM, kindly provided by Novartis (Basel, Switzerland) and ponatinib 1 to 50 nM). Cell survival evaluation was assessed by iPSC count at day 11.

### Cre-mediated vector excision

IPSC clones were transduced twice at an MOI of 100 with Cre-expressing adenovirus (kindly provided by AFM, Généthon). At day 7, iPSCs were dissociated into single cells with accutase (Stem Cell Technologies) and cloned by limiting dilution. Cre-lox excision of proviral reprogramming cassettes was determined in each subclone by PCR analysis. Primers used were: for OSK 1 detection: forward primer: GATGAACTGACCAGGCACTA and reverse primer: CTCGAGGGAATTCCGATAA; for MshP53 detection forward: TTCCGATCACGAGACTAG and reverse: GAGCAGAGCCCGGAGCGG.

### Hematopoietic differentiation of iPSCs

Hematopoietic differentiation of iPSCs was performed as described by Woods NB [15] et al with modifications [12]. Briefly, after embryonic bodies (EB) generation, to induce the mesodermal transition, newly generated EB were cultured on mitomycined OP9 feeder-cells (CRL-2749 from ATCC, Manassas, VA, USA) on Matrigel (BD Biosciences) for 12 additional days with partial medium change every day in a mesodermal specific medium [DMEM/F12, 15% FBS with the following cytokines: BMP4, low dose of VEGF, TPO, EPO, SCF, and Flt3L (all from PeproTech, at the concentrations described by Woods et al, [15]), with holotransferrine and ascorbic acid (from Sigma-Aldrich), and with PGE_2_ from Cayman Chemical]. From D14 to D21 of hematopoietic differentiation, the medium was changed to serum free expansion medium (Stem Cell Technologies) supplemented with TPO, EPO, SCF, Flt3L and PGE_2_ to promote the hematopoietic differentiation and HSC expansion. FACS analysis of CD34^+^ and CD45^+^ cells was performed at day 21 to evaluate the hematopoietic differentiation efficiency.

Single-cell suspensions of hematopoietic cells were plated in a methylcellulose-based medium of MethoCult H4435 (StemCell Technologies) (approximately 10^5^ cells) in 6-well plates. At day 14, colonies were observed by bright-field microscopy using a Nikon^TM^ ECLIPSE^TM^ Ti inverted microscope (Nikon) and captured with a digital sight camera and NIS-element^TM^ imaging software.

### Erythroid and myeloid differentiation

For erythroid and myeloid differentiation, we performed a 2-week protocol following hematopoietic differentiation. Briefly, cells were seeded in a 6-well low attachment plate with erythroid medium [Stem-alpha AE base (Stem Cell Technologies) supplemented with human plasma 5%, Epo 5 U/ml, SCF 50 ng/ml from Peprotech, holotransferrine 1 mg/ml, dexamethasone 10^−6^ M, insulin 20 ng/ml, β-mercapto-ethanol 10^−4^ M (Sigma Aldrich)] or myeloid medium [Stem-alpha (Stem Cell Technologies) supplemented with SCF (50 ng/ml), Flt3-L (50 ng/ml), TPO (50 ng/ml), GM-CSF (10 ng/mL), Il-3 (10 ng/mL), and Il-6 (5 ng/mL)]. FACS analysis of CD33^+^ and GPA^+^ cells was performed at day 15 to evaluate erythroid and myeloid differentiation efficiencies.

### Flow Cytometry

Cells were individualized from the differentiation cultures, collected and washed with PBS-HSA 1%. Cells were stained using phycoerythrin (PE) or FITC-conjugated anti-CD34, PECy5 or PE-conjugated anti-CD45, APC-conjugated anti-CD33, APC-conjugated anti-GpA, (all from BD, Franklin Lakes, NJ, USA).

For the apoptosis analysis, apoptotic adherent and non-adherent cells still present after hematopoietic differentiation were eliminated by Ficoll gradient. Live cells were plated on mitomycined OP9 in hematopoietic medium (Stem alpha-A complemented with Flt3L 50 ng/mL, SCF 20 ng/mL, TPO 50 ng/mL) with or without imatinib (5 µM for 24 h). The CD34^+^ cells were then analyzed for annexin-V binding after CD34^+^ gating (FITC Annexin-V Apoptosis detection kit, BD).

Cells were analyzed on a FACS (Canto II, flow cytometer BD, San Jose, CA, USA).

### Statistical Analysis

Results are expressed as mean ± SD or SEM as indicated in the legend figures. Statistical tests were performed with Student's tests. *p*<0.05 was considered statistically significant.

## Results

### Generation and characterization of human iPSCs from normal and CML-derived CD34^+^ cells

**Figure 1:**
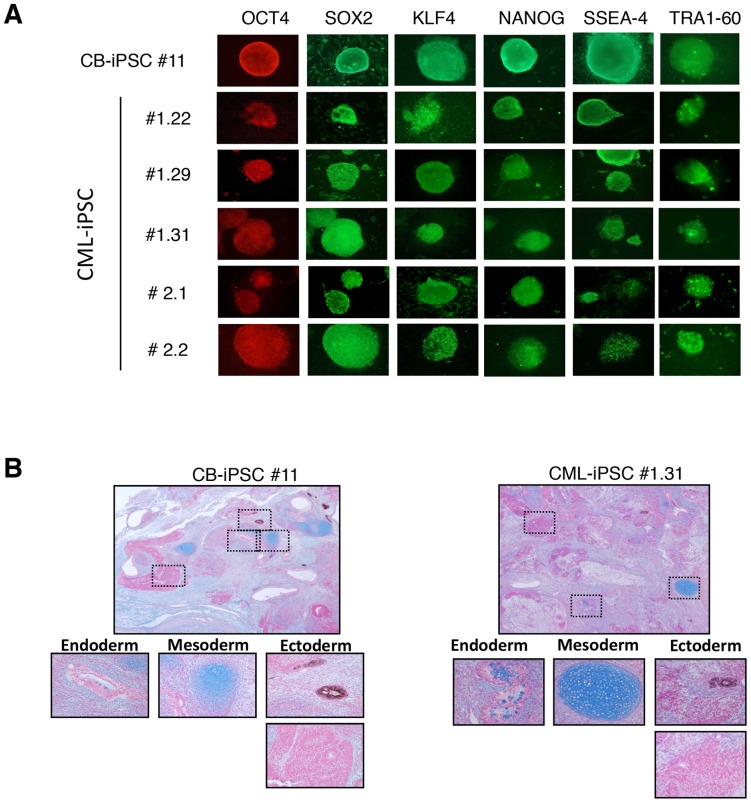
Characterization of iPSC clones. (**A**) Representative immunofluorescence of pluripotency markers in human iPSC clones derived from CD34^+^ CB cells (CB-iPSC #11) and CD34^+^ from CML first patient (CML-iPSCs #1.22, #1.24 and #1.31) and from CML second patient (#2.1 and #2.2), staining with anti-OCT4, anti-SOX2, anti-KLF4, anti-NANOG, anti-SSEA-4 and anti-TRA1-60. MEFs surrounding human iPSCs served as a negative control for immunofluorescence (magnification x100 or x200). (**B**) Representative alcian blue staining of histological sections of teratoma derived from human CB-iPSC #11 and CML-iPSC #1.31 encompassing tissues with all three germ layers (magnification x25 and x200).

We have generated a total of ten iPSCs clones characterized (two CB-iPSCs, six CML-iPSCs from the CML patient #1.X and two CML-iPSCs from the CML patient #2.X) (Fig 1A). Cells from the two CML patients were collected at diagnosis, in chronic phase. Thereafter, these patients had good response to imatinib treatment (Major Molecular Response after 6-month-imatinib-treatment). All the harvested colonies demonstrated the typical characteristics of pluripotent stem cells: morphology similar to that of human ES cells, strong alkaline phosphatase activity and expression of pluripotent stem cell markers as evidenced by immunocytochemistry such as OCT3/4, SOX2, KLF4, NANOG, SSEA-4 and TRA1-60 (Fig 1A). iPSC xenografts into immunodeficient NOD-scid IL2Rgamma^null^ mice (NSG) resulted in the formation of teratomas *composed of derivatives from* all 3 embryonic germ layers demonstrating in vivo pluripotency of the iPSC clones (Fig 1B).

**Figure 2:**
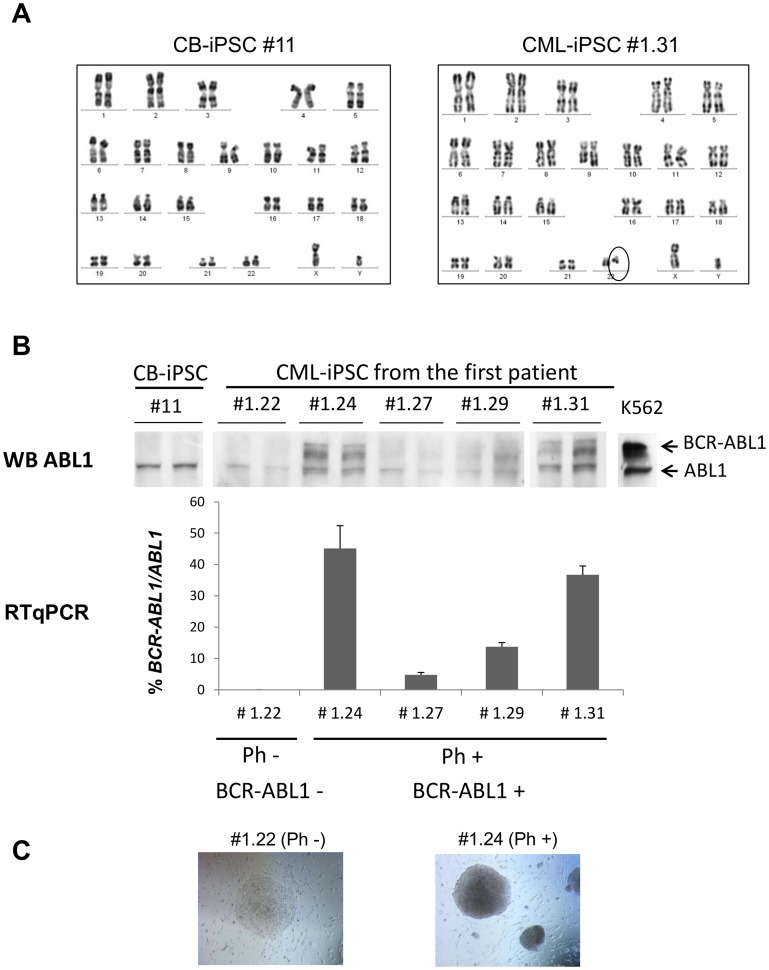
BCR-ABL1 expression in CML-iPSCs. (**A**) Representative karyotype analysis of human CB-iPSC clones #11 and CML-iPSC #1.31 (Philadelphia chromosome positive surrounded). (**B**) Western-blot using anti-ABL1 antibody (upper panel, 2 lines per clone) and RT-qPCR analysis (lower panel) of *BCR-ABL1* expression from 5 CML-iPSCs from the first CML patient. CB-iPSC #11 was used as a negative control and K562 as a positive control for western-blot analysis of BCR-ABL1 expression. Bars graph showing mean + SD of triplicate. (**C**) iPSC morphology (magnification ×40).

Karyotypic analyses revealed that in CML-iPSCs, the chromosome Ph was present in all CML-iPSCs (Ph+) except the #1.22 (Ph-) (Fig 2A). The absence of translocation between the chromosomes 9 and 22 in the CML-iPSC #1.22 was confirmed by the absence of the BCR-ABL1 fusion protein and BCR-ABL1 transcript (Fig 2B). The CML-iPSC #1.22 (Ph-) was an interesting clone illustrating the well-known presence of Ph- cells at diagnosis in CML and used as in internal control in our study. Among the 5 Ph+ CML-iPSCs characterized from the patient #1.X, we observed heterogeneous BCR-ABL1 expression and transcript levels (Fig 2B). The transcript level was significantly different between clones except between clone #1.24 versus clone #1.31.

We noticed that Ph+ CML-iPSC colonies were different from the Ph- colonies. They were sharp-edged like regular ESCs but less flat, and the colonies appeared more aggregated (Fig 2C). Moreover, after unicellular dissociation they displayed higher viability than the Ph- iPSC colonies, including the clone *#*1.22 from the CML patient 1.

### Absence of TKI toxicity on CML-iPSCs

**Figure 3:**
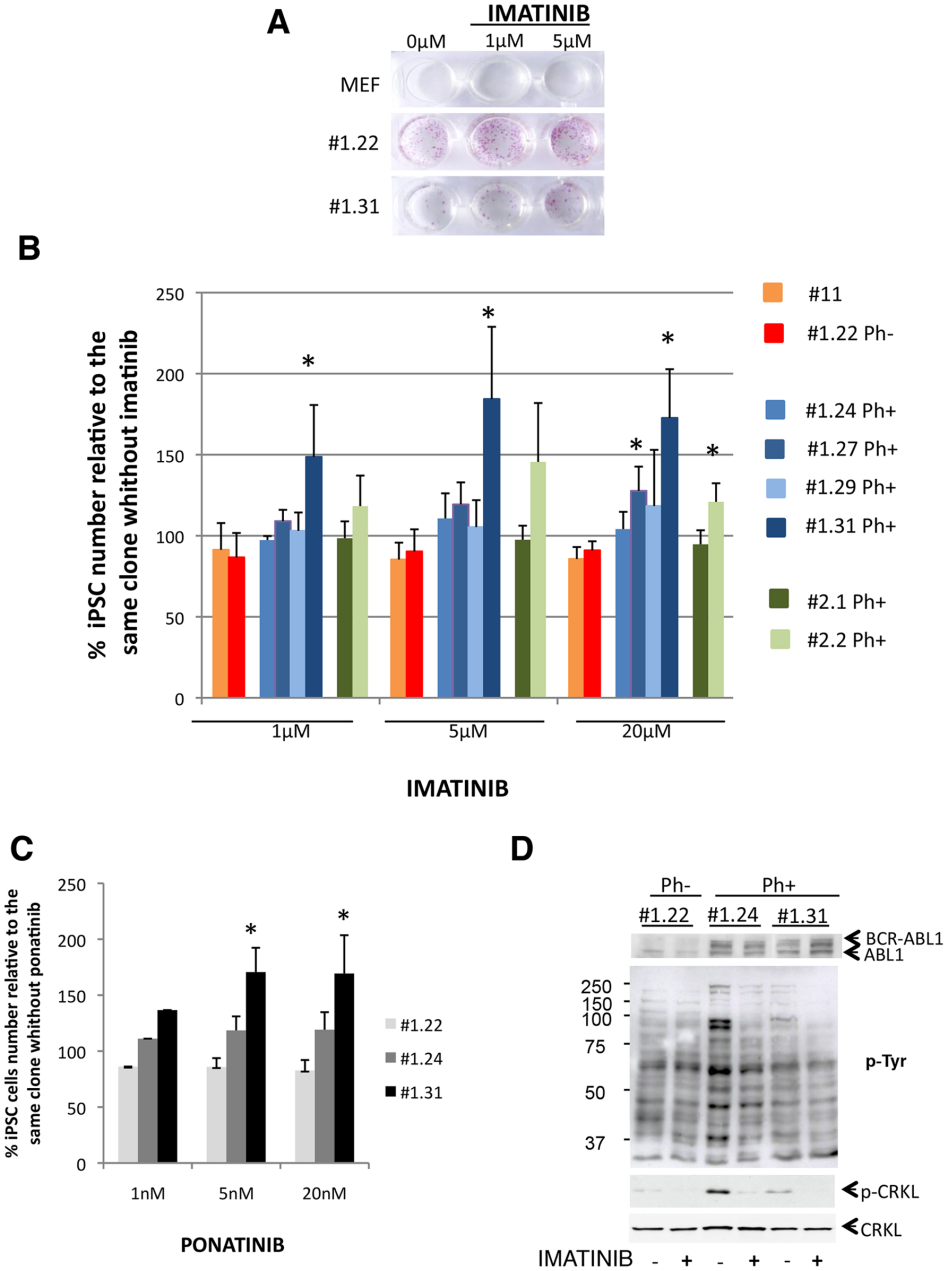
BCR-ABL1 independent proliferation. (**A**) Dose-effect of imatinib exposure (0–5 µM) for 6 days on CML-iPSC clones #1.22 and #1.31. Colony frequency is evaluated by alkaline phosphatase staining conducted at day 6. (**B**) Dose-effect of imatinib exposure for 6 days on iPSCs survival. iPSCs counts were conducted at day 6 and are expressed as percentages relative to same iPSC . Mean +/− SD n = 3, *: p<0.05 versus clone #1.22 with the same exposure. (**C**) Dose-effect of ponatinib exposure for 6 days on CML-iPSC clones (#1.22 Ph-, #1.24 and #1. 31 Ph+) survival. iPSCs counts are conducted at day 6 and expressed as percentages relative to same iPSC without TKI. Mean +/- SD, n = 3. * p <0.05 vs iPSC #1.22 (internal control Ph-) at the same TKI exposure. (**D**) Western-blot analysis of ABL, phosphotyr (p-Tyr) pattern, CRKL and phosphoCRKL (p-CRKL) in CML-iPSCs in absence (−) or presence (+) of imatinib (20 µM) for 48 h.

In order to determine the CML-iPSC sensitivity to TKI, we initially performed a preliminary experiment to determine the imatinib effect on the control CML-iPSC #1.22 (Ph-) and the CML-iPSC #1.31 (Ph+), at 1 and 5 µM for 6 days. The iPSC colony number was determined after phosphatase alkaline staining. We did not observe imatinib-induced toxicity on either CML-iPSC clones (Fig 3A). To test the possibility that the doses used were insufficient to induce toxicity on CML-iPSCs Ph+, imatinib concentrations were increased up to 20 µM on 2 iPSC clones Ph- (CB-iPSC #11 and CML-iPSC #1.22) and 6 CML-iPSC clones Ph+ (#1.24, #1.27, #1.29, #1.31, #2.1 and #2.2). All tested iPSC clones were resistant to imatinib treatment, even at the highest dose (20 µM) and after a long exposure to imatinib (6 days) (Fig 3B, Ph- clones in red/orange, Ph+ clones from CML patient #1 in blue, Ph+ clones from CML patient #2 in green). The same results were obtained with ponatinib, a third generation TKI (Fig 3C). Moreover, surprisingly, two Ph+ CML-iPSC clones (#1.31 and #2.2) grew even faster in presence of high doses of imatinib and ponatinib (Fig 3B and 3C).

### BCR-ABL1 independency of CML-iPSCs

**Figure 4:**
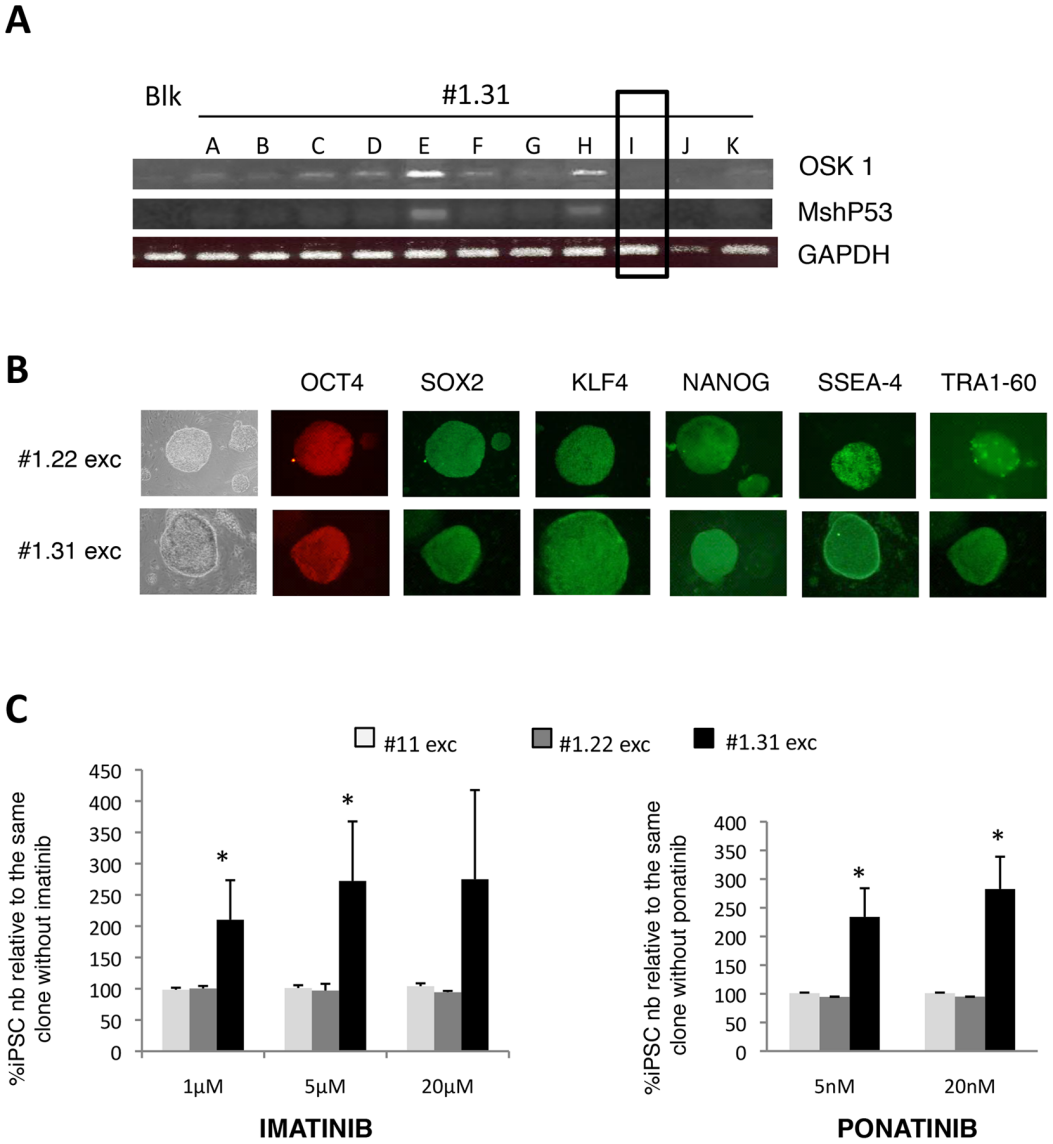
Transgene independence of CML-iPSCs survival in presence of TKI. (**A**) PCR for the integrated vectors OSK 1 and MshP53 in 11 subclones of CML-iPSC #1.31 pretreated with CRE adenovirus. Generation of transgene-free subclone CML-iPSC #1.31i: excision of the 2 vectors. (**B**) Immunohistochemistry of pluripotency markers: OCT4, SOX2, KLF4, NANOG, SSEA-4 and TRA1-60 in human transgene-free iPSC subclones (after excision) derived from CD34^+^ from CML patient (#1.22 exc and #1.31 exc) (**C**) Dose-effect of TKI exposure (with imatinib (left panel) or ponatinib (right panel)) for 6 days on human excised CML-iPSCs (# 1.22, #1.31) and CB-iPSC (#11) subclones survival. iPSCs counts are conducted at day 6 and expressed as percentages relative to same iPSC clone without TKI. Mean ± SD of triplicate.

To explain the absence of toxicity of the TKI, we first hypothesized that the TKI did not inhibit the BCR-ABL1 activity (by BCR-ABL1 kinase domain mutations or drug efflux for example). To investigate this point, we performed a western-blot analysis to determine the level of total phosphotyrosines and phospho-CRK-like protein (CRKL), a specific substrate of BCR-ABL1. We showed that imatinib (20 µM) decreased the total phosphotyrosine level and abrogated most of the phospho-CRK-like protein (CRKL) in CML-iPSCs Ph+ (Fig 3D). Despite the absence of imatinib-induced toxicity, these results demonstrated that this drug efficiently inhibited its target i.e. the BCR-ABL1 activity. However, it was possible that the persistence of exogenous reprogramming factors in CML-iPSCs could interfere with their response to TKI. To address this issue, we created iPSCs devoid of exogenous reprogramming factors. This was possible because the transgenic cassettes were flanked by the loxP sites, and excisable by adenovirus-mediated CRE recombinase. After subcloning of the three iPSCs (CB-iPSC #11, CML-iPSC Ph- #1.22 and CML-iPSC Ph+ #1.31), DNA-PCR analysis was performed to select the rare clones with excision of both reprogramming cassettes (Fig 4A). Immunocytochemistry for pluripotency markers (fig 4B) and RTqPCR of pluripotency genes (data not shown) confirmed that the excised subclones were still pluripotent. Neither imatinib nor ponatinib, even at the highest concentrations, induced toxicity on the excised Ph+ CML-iPSCs (Fig 4C). Interestingly these data demonstrate that CML-iPSC survival is independent of the oncogenes possibly supporting their growth.

**Figure 5:**
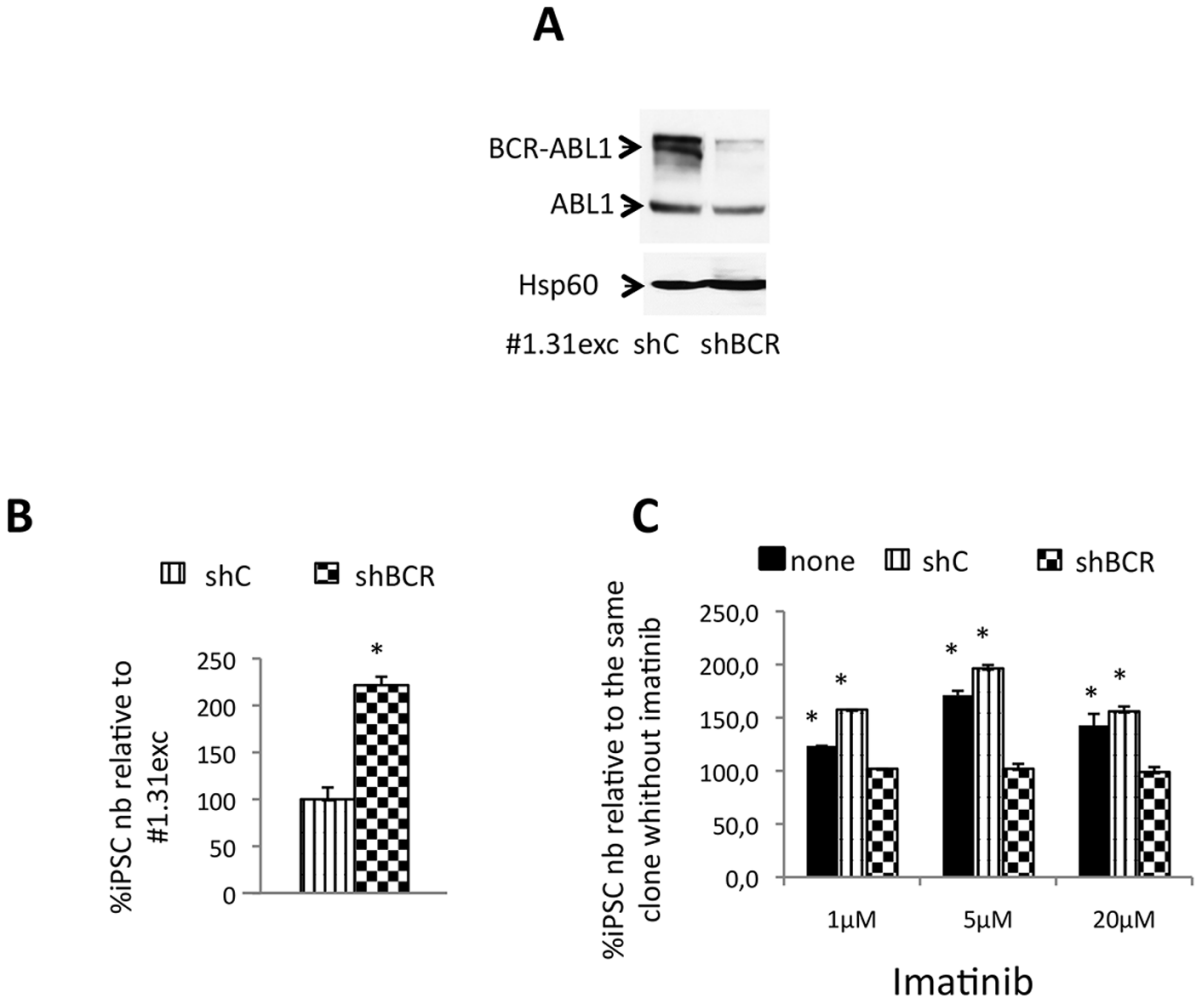
Effect of shRNA against BCR-ABL1 on CML-iPSC #1.31 clone proliferation. (**A**) Western blot analysis of BCR-ABL1 and ABL expression in CML-iPSC #1.31 with shRNA control (shC) and with shRNA against BCR-ABL1 (shBCR). (**B**) Left panel: Proliferation of CML-iPSC (#1.31) with shC or shBCR. iPSCs counts at day 6 expressed as percentages relative to same iPSC (CML-iPSC #1.31) with shC. Mean +/− SD, n = 3. Right panel: Dose-effect of imatinib exposure for 6 days on iPSCs (CML-iPSC #1.31, CML-iPSC #1.31 with shC or with sh BCR). iPSCs counts are conducted at day 6 and expressed as percentages relative to same iPSC without TKI. Mean ± SD, n = 3.

To further explore the particular behavior of CML-iPSC #1.31 in the presence of TKI, we explored the BCR-ABL1 implication in this process. This TKI effect could be due to the specific BCR-ABL1 kinase inhibition or to an off-target effect. Thus, we transduced the CML-iPSC #1.31 with a lentiviral vector containing a shRNA directed against the BCR-ABL1 junction or with a control shRNA. This resulted in a strong down-regulation of BCR-ABL1 expression (Fig 5A). ShRNA BCR-ABL1 induced the proliferation on this particular clone (Fig 5B) in a similar way than after imatinib exposure. When this clone (#1.31) was transduced with the shRNA BCR-ABL1, imatinib did not induce proliferation, like in control Ph- iPSC clones (Fig 5C). This result confirms that TKI induced-proliferation in this clone was BCR-ABL1 dependent. Thus, the particular behavior of the CML-iPSC #1.31 was specifically dependent of BCR-ABL1 activity inhibition.

### Reduced hematopoietic differentiation of CML-iPSC clones compared to control iPSCs

**Figure 6:**
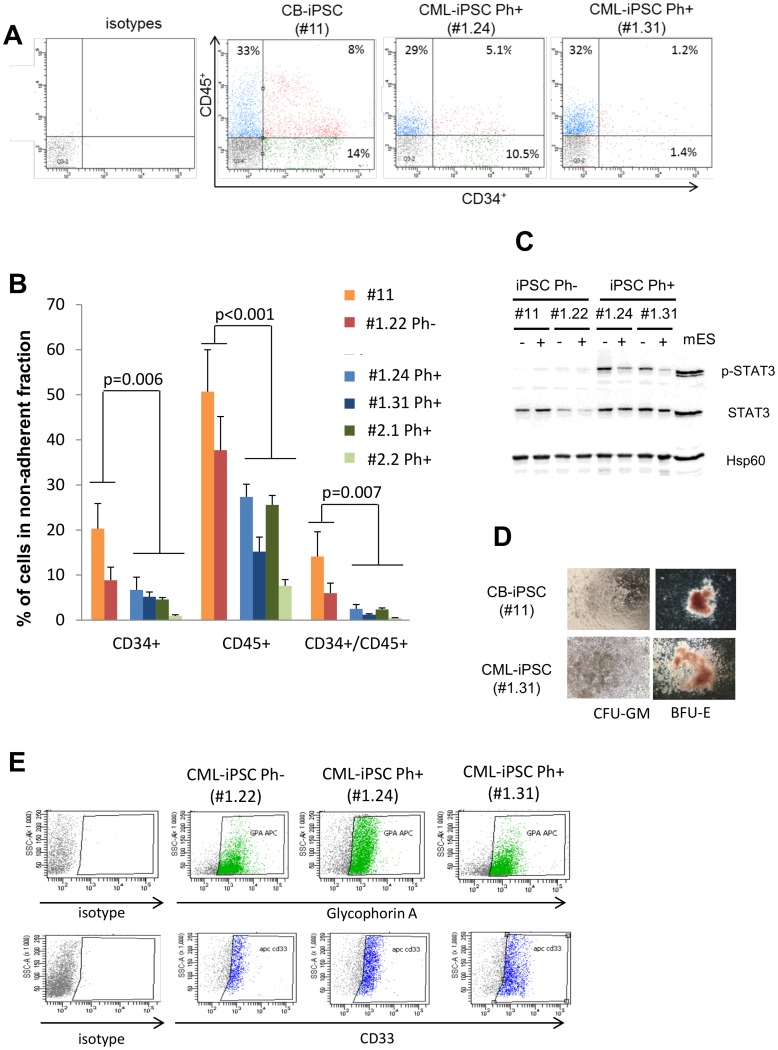
Hematopoietic differentiation of CML-iPSCs. (**A**) Representative FACS analysis of CD45^+^ and CD34^+^ cells obtained from CB-iPSC #11, CML-iPSC #1.24 and CML-iPSC #1.31, after hematopoietic differentiation (at day 21), in non-adherent fraction. (**B**) Bar graphs showing average percentages of CD34^+^, CD45^+^ and CD34^+^/CD45^+^ cells obtained in non-adherent fractions at day 21 of hematopoietic differentiation (n = 5 independent experiments, mean ± SEM). (**C**) Western-blot analysis of total STAT3, phosphorylated STAT3 (p-STAT3) in Ph^-^ iPSC (CB-iPSC #11 and CML-iPSC clones #1.22) and in Ph^+^ iPSCs #1.24 and #1.31 in absence (−) or presence (+) of imatinib (20 µM) for 48 h. Murine embryonic stem cell extract (mES) in presence of LIF is used as positive control for STAT3 and pSTAT expression. (**D**) Bright field microscopy of colony forming units in methylcellulose medium (granulo-monocytic (CFU-GM) and erythroid (BFU-E)) obtained by hematopoietic cells derived from excised CB-iPSC #11 (upper panel) or Ph^+^ CML-iPSC #1.31 (lower panel) (magnification x100). (**E**) FACS analysis of glycophorin A^+^ and CD33^+^ cells obtained from Ph^−^ iPSC #1.22, Ph^+^ CML-iPSCs #1.24 and #1.31.

To generate hematopoietic cells including hematopoietic progenitors and stem cells (HSPCs), we used the highly efficient optimized three-week protocol described by Woods et al with some modifications (days 1 to 21) [12]. CD34^+^ hematopoietic cells were obtained from the CB-iPSC #11, the Ph- CML-iPSC #1.22, and the Ph+ CML-iPSCs (Fig 6A and 6B) with various efficiencies. We observed in non-adherent compartments high yields from the CB-iPSC #11 and from the CML-iPSC #1.22 Ph-: the mean percentages of hematopoietic cells generated were equal to 50.7% and 37.7% for CD45^+^ cells; 20.3% and 9% for CD34^+^ cells; 14.1% and 6.1% for CD34^+^/CD45^+^ cells, for the CB-iPSC #11 and CML-iPSC #22 respectively (Fig 6B). By contrast, lower yields were obtained for the four CML-iPSCs Ph+ (#1.24 and #1.31 from the first CML patient and (#2.1 and #2.2 from the second one), compared to the two Ph- clones: the mean percentages of CD45^+^ cells generated was equal to 15% for the Ph+ versus 41% for the Ph- clones (p<0.001), 4.2% versus 13.3% (p = 0.006) for the CD34^+^ cells and 1.2% versus 9.1% for the CD34^+^/ CD45^+^ cells (Fig 6B).

In murine embryonic stem cells (mESCs), the pluripotency is maintained by the signaling pathway LIF/gp130/p-STAT3. Coppo et al demonstrated the inhibitory role of high p-STAT3 levels in the hematopoietic differentiation of mESCs expressing BCR-ABL1 [16]. Western-blot analysis revealed high p-STAT3 levels in CML-iPSCs Ph+ (#1.24 and #1.31 from the first CML patient (Fig 6C), and #2.1 and #2.2 from the second one (data not shown) but p-STAT3 was undetectable or evidenced at very low levels in iPSCs Ph- (#11 and #1.22) (Fig 6C). Interestingly, like in mESCs, high levels of p-STAT3 were observed in iPSC with low capability of hematopoietic differentiation and iPSC displaying the highest percentages of hematopoietic cell differentiation lack p-STAT3. In addition, imatinib exposure reduced its phosphorylation (Fig 6C). These data suggest that in human CML-iPSCs Ph+, BCR-ABL1 phosphorylates STAT-3 and this could limit the hematopoietic differentiation.

We noticed variable yields of generated CD34^+^/CD45^+^ hematopoietic cells from Ph+ clones from the same patient (patient #1 : 2.5% versus 0.9% (respectively for #1.24 and #1.31, p = 0.04) and patient #2: 2.4% versus 0.5% (respectively for #2.1 and #2.2, p = 0.002).

However, all clones were able to produce CFU (colony forming units) in methylcellulose (Fig 6D). Moreover, we induced liquid erythroid and myeloid differentiations. FACS analysis showed the presence of myeloid cells (CD33^+^) and erythroid cells (GPA^+^) at day 14, confirming the differentiation capability of the CD34^+^ hematopoietic progenitors derived from the CML-iPSCs (Fig 6E).

### Sensitivity to TKI of hematopoietic progenitors derived from the CML-iPSCs

**Figure 7:**
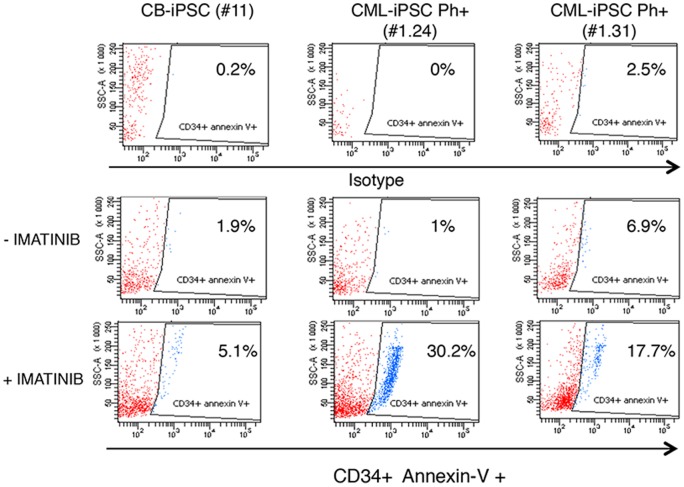
Partial restoration of TKI-sensitivity of CD34*^+^* hematopoietic progenitors derived from CML-iPSCs. Partial restoration of sensitivity to TKI of CD34^+^ hematopoietic progenitors derived from CML-iPSCs. Apoptosis in untreated versus imatinib cultures (5 µM, 24 h) was evaluated after annexin-V staining by FACS analysis, in CD34^+^ cells derived from CB-iPSC #11, CML-iPSCs #1.24 and #1.31.

Given that CML-iPSCs Ph+ lost their BCR-ABL1 dependency, we evaluated whether after hematopoietic re-differentiation, CD34^+^ hematopoietic progenitors derived from CML-iPSC Ph+ recovered their BCR-ABL1 addiction revealed by restored sensitivity to TKI. To test TKI effect, we salvaged CD34^+^ cells derived from the CB-iPSCs and CML-iPSCs and incubated them with or without imatinib (5 µM) in hematopoietic medium. After 24 h, increased apoptosis was observed for imatinib-treated cultures of CD34^+^ cells derived from the Ph+ CML-iPSCs (Fig 7). The percentages of CD34^+^/annexin V^+^ cells specifically induced by imatinib was of 29.2% for the CML-iPSC #1.24 and 10.8% for the CML-iPSC #1.31 indicating partial restoration of imatinib sensitivity in CML-derived CD34^+^ cells.

## Discussion

In this work, we obtained iPSCs from CML patients. The reprogramming efficiency of peripheral CML CD34^+^ cells was lower than that of CB-CD34^+^ control cells (0.01% vs 0.1%, respectively), and delayed (21 days vs 14 days). This result could be accounted for the fact that cancer-specific genetic lesions might be a hindrance for reprogramming cancer cells illustrated by the rare cases of successful cancer cells reprogramming reported [17].

Interestingly, despite Ph+ CML-iPSC had all iPSC characteristics (pluripotent markers, teratoma capability), we observed particular morphology with sharp-edged like ESCs but less flat, more aggregated colonies and more tolerant to passaging as single cells than Ph- iPSC, including the clone *#*1.22 from CML patient. This analogy with mESC, already observed by Hanna J et al in human iPSC in presence of LIF [18], could be explained by the presence of p-STAT3, induced by BCR-ABL1 in our clones, and by LIF/gp130/JAK signaling pathway in mESC.

Understanding the mechanisms leading to TKI resistance of the LSCs in CML is a critical issue but is limited by availability of cells from patients. Similar to previously published papers with iPSCs derived from CML cell lines [19] and more recently from CML primary cells [20], [21], we found that CML-iPSCs generated expressed BCR-ABL1, but were resistant to imatinib, even after Crkl phosphorylation inhibition. Moreover, we showed that blood cells could be generated from CML-iPSCs, with partial restoration of TKI sensitivity.

For the first time, in this work, we tested TKI sensitivity and hematopoietic differentiation of several clones per patient. By establishing several independent clones per patient, we generated an iPSC clone from the residual normal cells of a CML patient which became an ideal normal control. Moreover, we were able to observe various behavior of the Ph+ iPSCs obtained from the same CML patients, in terms of BCR-ABL1 pattern, sensitivity to imatinib and hematopoietic differentiation. We cannot rule out that these variations could result from heterogeneity of iPSCs reprogramming, as recently published by Winkler et al [22]. To assess specific heterogeneity of hematopoietic differentiation from the CML-iPSC obtained from the same CML patient, it will be necessary to study more control iPSC and CML-derived iPSC clones. However, these results pointed out the necessity of studying multiple clones when using iPSCs to model disease, which is in total agreement with the present results. However, it is also likely that this variability may reflect of LSC heterogeneity at diagnosis. Indeed, a mathematical model proposed a higher probability of several leukemic clones with different growth characteristics instead of the presence of a predominant clone at the start of the treatment [23], [24], which is illustrated here, because we showed clonal diversity in iPSCs clones obtained from the same patient.

We did not limit our study to imatinib-resistance and used in addition the new highly efficient pan BCR-ABL1 inhibitor, ponatinib, and a shRNA against BCR-ABL1. We observed the same resistance of the iPSC clones. Moreover, by using two excisable lentiviral vectors, and studying TKI sensitivity with and without reprogramming cassettes, we demonstrated that the survival of the CML-iPSC clones was independent of the reprogramming factors.

Altogether, these data support that CML-iPSCs survival is independent of the BCR-ABL1 kinase activity at this pluripotent stage, possibly by specific signalling pathways of survival. This phenomenon is in agreement with the TKI resistance of primitive LSCs from CML, despite the kinase inhibition [6], [7].

We also showed that blood cells could be generated from CML-iPSCs. However, we notice that Ph+ CML-iPSC hematopoietic differentiation was reduced although reprogramming cassettes were excised [25]. Our data suggest that, as in mESCs [16], STAT3 is phosphorylated by BCR-ABL1, and could be in the partial inhibition process. Extended mechanistic analyses will be crucial to confirm the p-STAT3 pathway implication in inhibiting hematopoietic differentiation of the Ph+ CML-iPSCs.

Among the Ph+ clones, hematopoietic differentiation of two clones (*#*1.31 and *#*2.2) was particularly limited. However, neither p-STAT3 nor BCR-ABL1 levels were higher in these clones than in the other Ph+ clones with higher differentiation yields. Interestingly, they are the clones which paradoxically proliferated in presence of TKI (imatinib and ponatinib, even at high dose). For these particular clones, BCR-ABL1 seemed to actually slowdown cell growth as previously observed in imatinib-resistant cell lines [26]. A full characterization of these two clones (transcriptome and miRNome) will be necessary to discover signaling pathway implicated in this paradoxical behavior in presence of TKI. The next step will be to investigate whether primary LCSs activate the same pathways leading to residual disease.

In this study, we exemplified that CML-iPSCs can be used to study the mechanisms responsible for LSC survival following TKI therapy and are a promising tool for testing new therapeutics achieving the full destruction of LSC reservoirs for a permanent cure to CML patients. Despite the fact that the CML is considered as a unique and simple cancer model with a putative “one step” molecular hit driving the leukemic cells, it is undoubtedly a heterogeneous disease. The subset of patients with molecular remission leading to treatment cessation is itself heterogeneous as exemplified by the variable sequence of events occurring after imatinib cessation in CML patients.
